# Supplementary figures and images for: Transcription factor regulation of eQTL activity across individuals and tissues
Source: PLoS Genet. 2022 Jan 31;18(1):e1009719. doi: 10.1371/journal.pgen.1009719 (PMC8830792; doi:10.1371/journal.pgen.1009719)

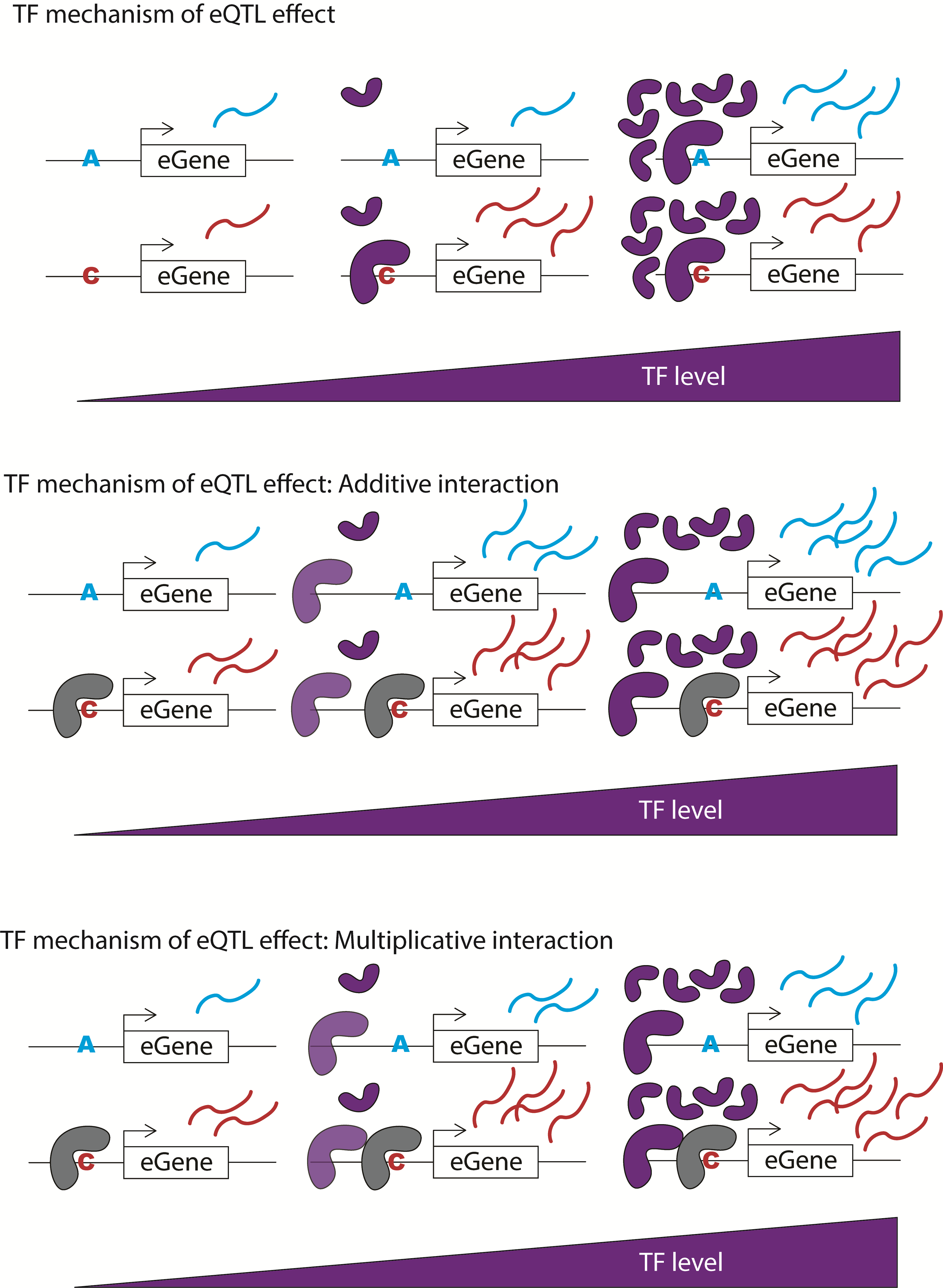

Supplement: S1 Fig — (Top) An eQTL variant increases the affinity of the purple TF, resulting in observable eQTL effects at mid-range levels of purple TF. (Middle) An eQTL variant disrupts the affinity of the gray TF, while the purple TF binds to a different region of the cis-regulatory region which interacts additively with the region where the gray TF binds. The eQTL effect will be observable at low purple TF levels, but may become overpowered and unobservable if purple TF levels increase and expression generated by the purple TF locus exceeds that generated by the gray TF locus. (Bottom) An eQTL variant disrupts the affinity of the gray TF, while the purple TF binds binds to the gene’s cis-regulatory region and interacts multiplicatively with the region where the gray TF binds. The eQTL effect should remain constant at all purple TF levels. (TIF) [file pgen.1009719.s001.tif]

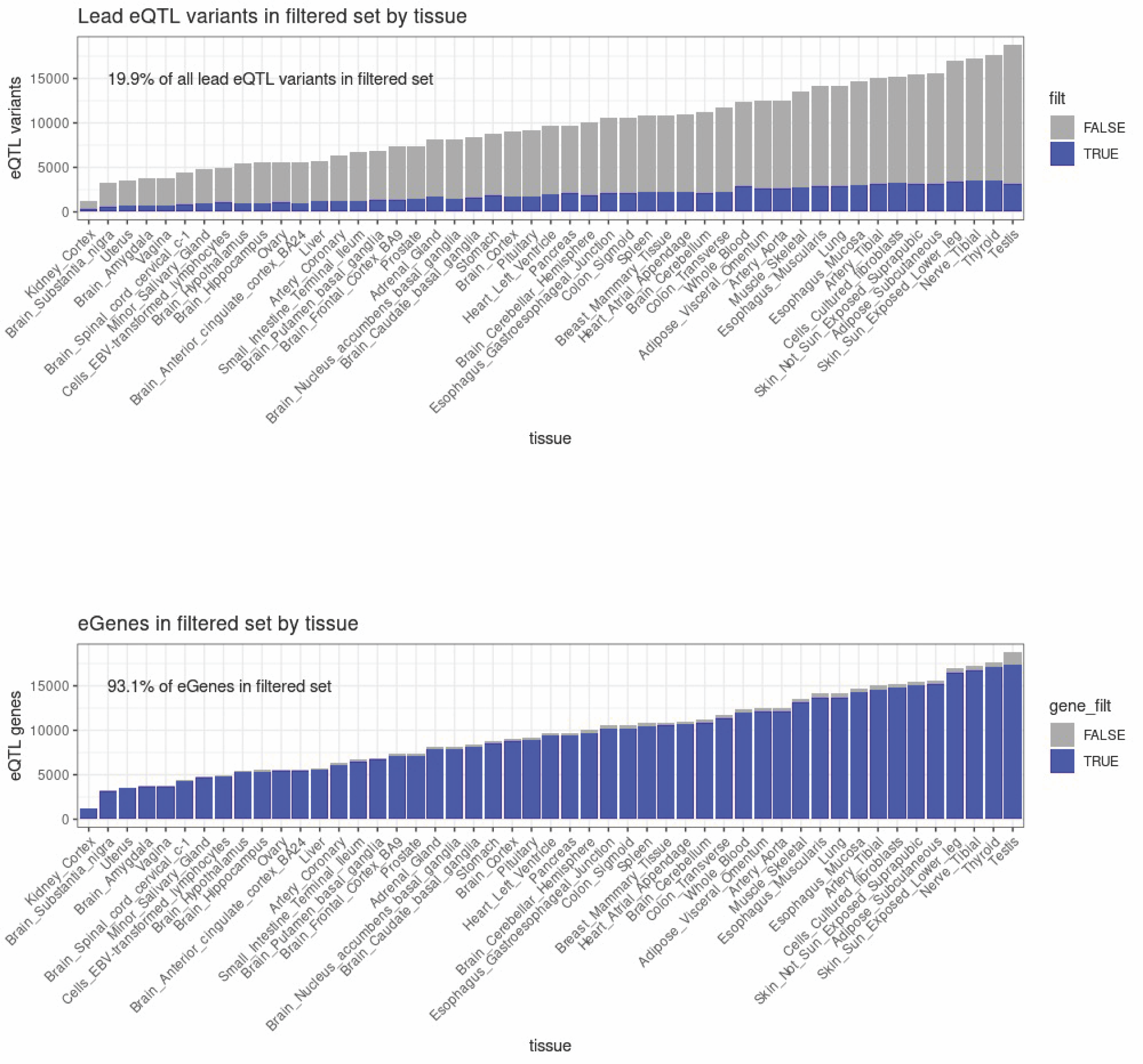

Supplement: S2 Fig — (Top) The presence of lead eQTLs that are found in the filtered eVariant-eGene set are plotted by tissue. The inset displays the total percent of lead tissue eQTLs in the filtered set. (Bottom) The presence of eGenes that are found in the filtered eVariant-eGene set are plotted by tissue. The inset displays the percent of all eGenes that are in the filtered set. (TIF) [file pgen.1009719.s002.tif]

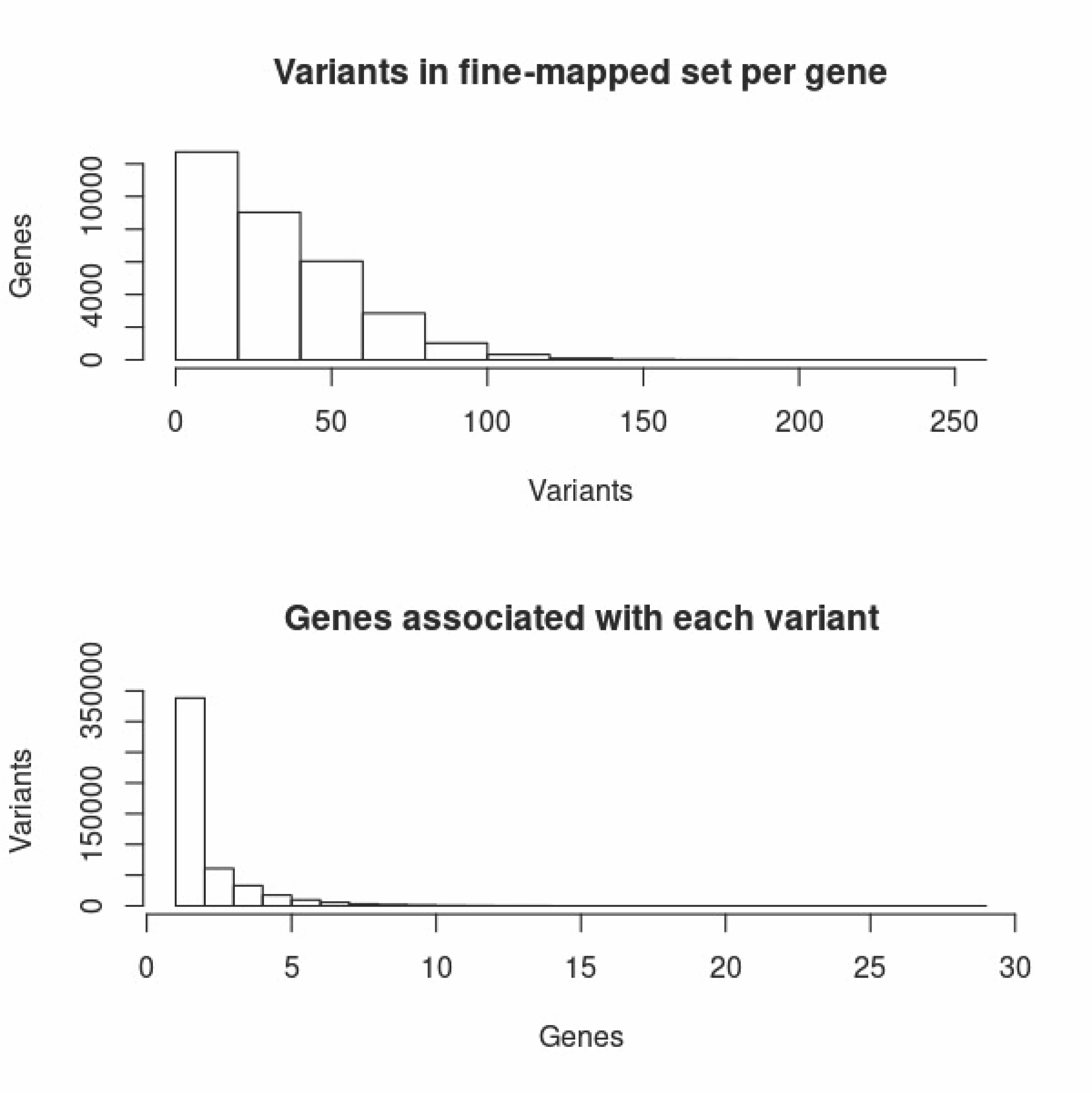

Supplement: S3 Fig — Caviar fine-mapped variants were overlapped with TF ChIPseq and motif variants. The number of resulting fine-mapped variants per gene (top) and genes per variant (bottom) are displayed. (TIF) [file pgen.1009719.s003.tif]

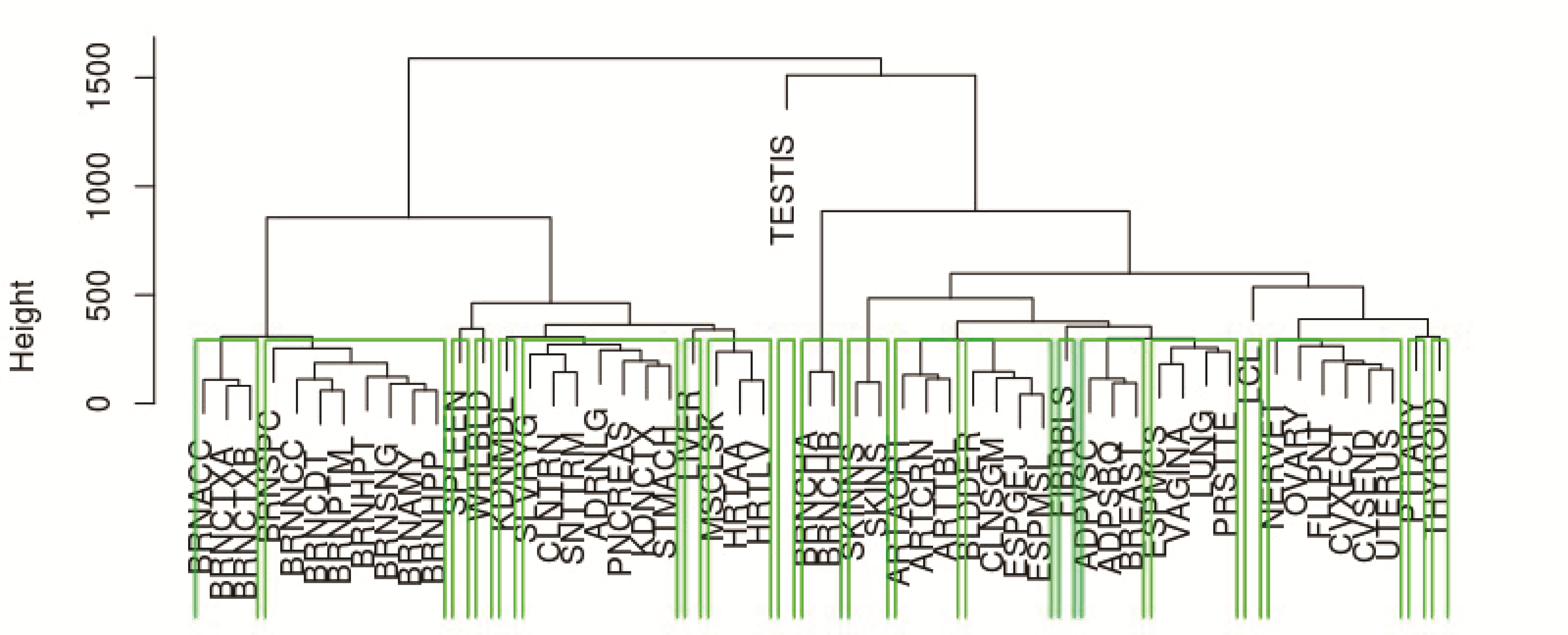

Supplement: S4 Fig — We clustered tissues based on median TPM across all genes using Euclidean distances and Ward.D clustering, cut the resulting tree to generate twenty clusters (green boxes). The tissue with the largest sample size was selected from each cluster. (TIF) [file pgen.1009719.s004.tif]

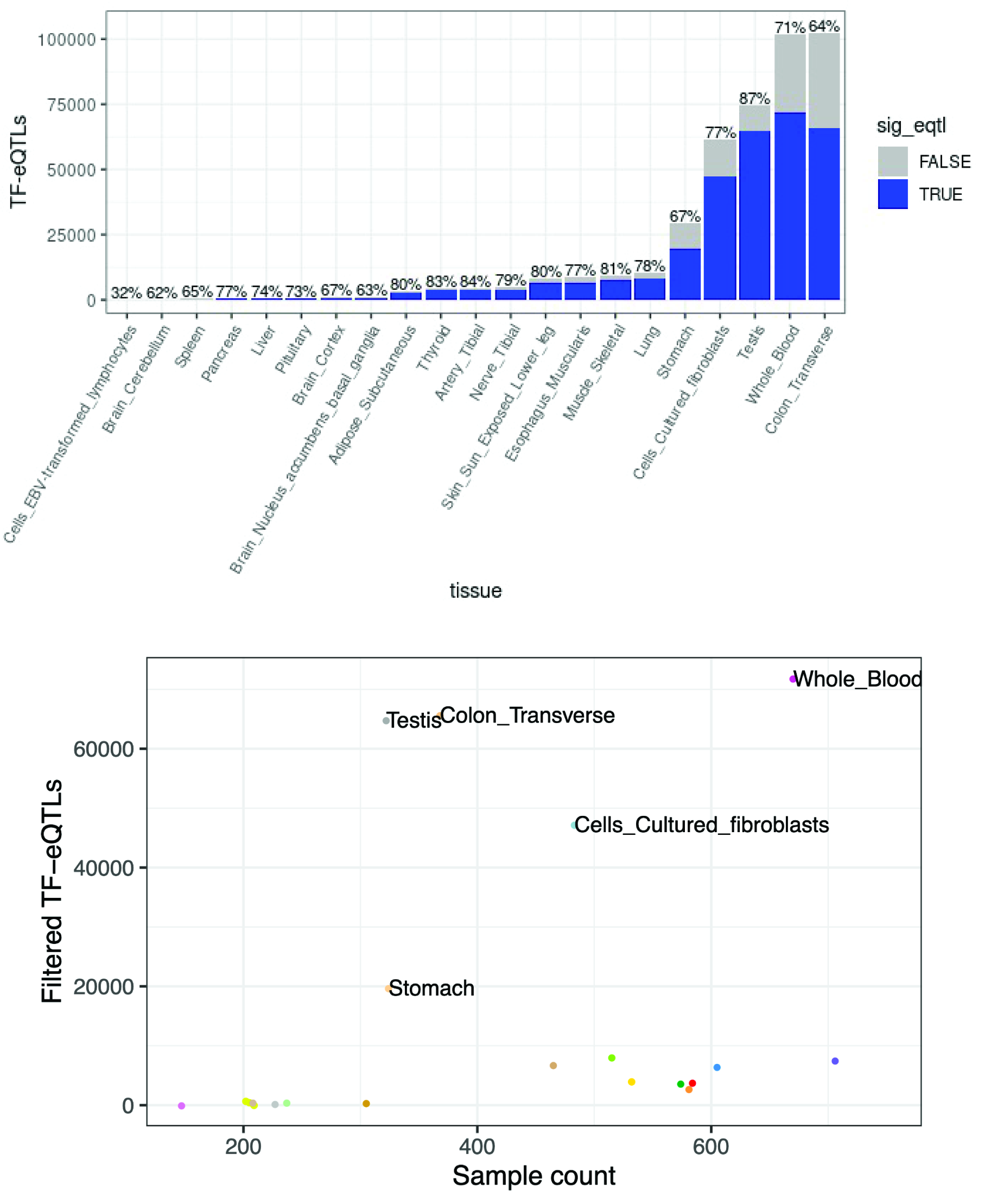

Supplement: S5 Fig — 5% FDR TF-eQTLs per tissue, colored by whether or not top TF-eQTL variant was significantly associated with gene expression in that tissue. Only TF-eQTL variants with a significant eQTL were retained for further analysis. (Bottom) Tissues plotted by number of TF-eQTLs vs. tissue sample size. Outlier tissues are labeled. (TIF) [file pgen.1009719.s005.tif]

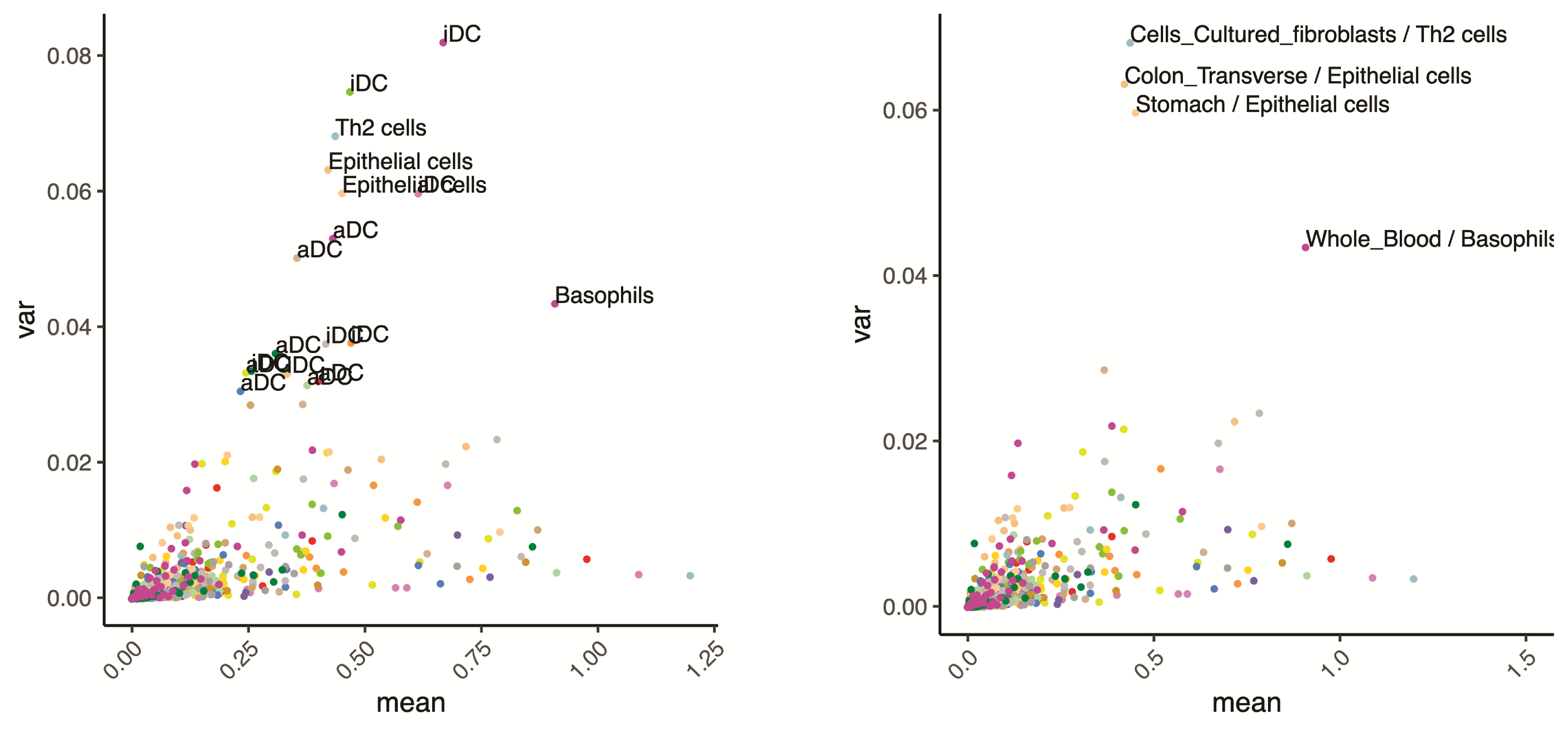

Supplement: S6 Fig — Cell type enrichments were calculated in silico using XCell, and the mean/variance of each cell type in each tissue was calculated. Both plots show cell type variance vs. mean per tissue (dot color). aDC and iDC estimates frequently had large variance (left), thus they were removed (right). Four tissues remained with large cell type variance: fibroblasts, colon, stomach, and blood. (TIF) [file pgen.1009719.s006.tif]

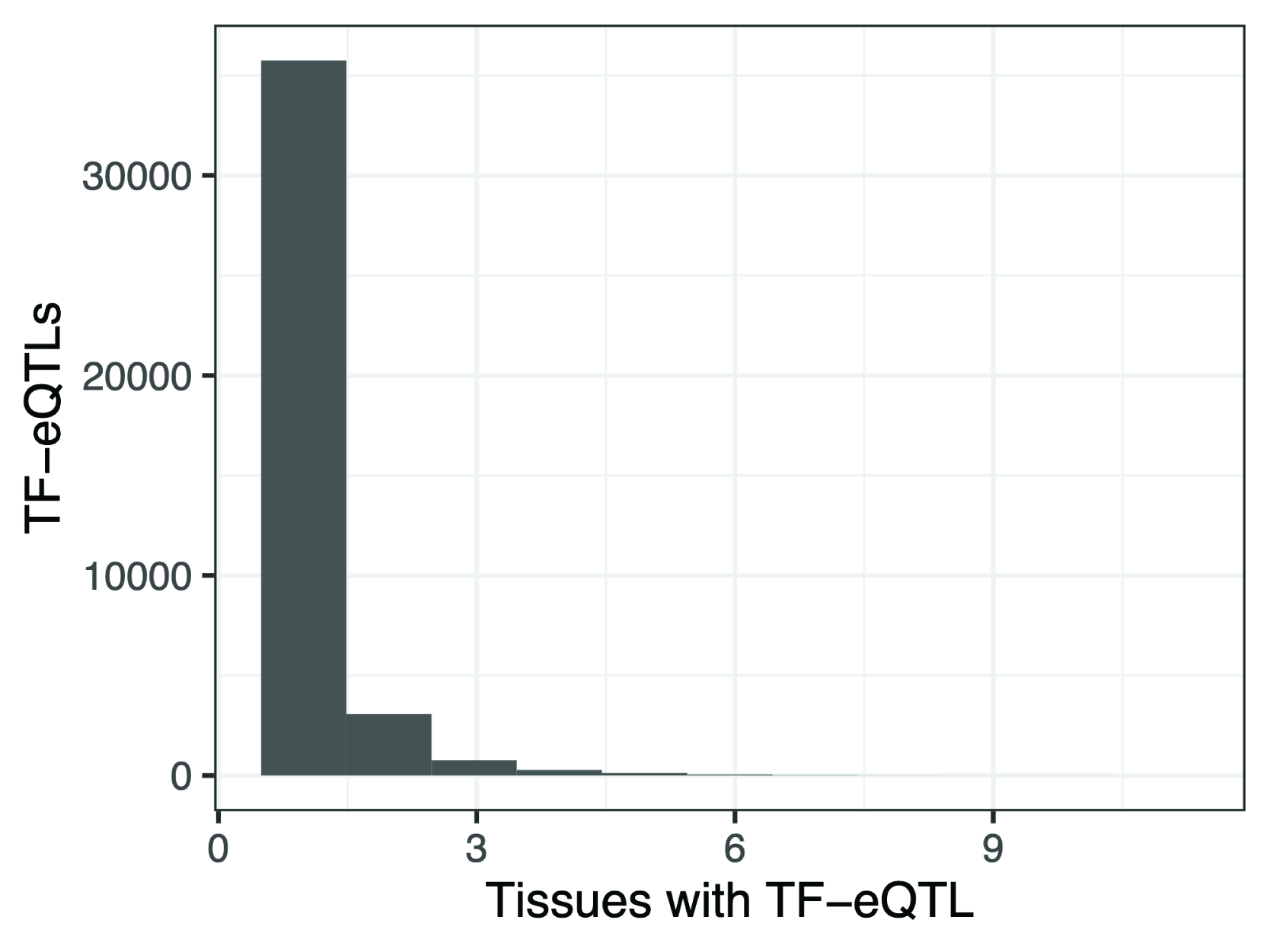

Supplement: S7 Fig — The majority of TF-eQTLs were seen in one tissue only, though 4318/40065 (10.8%) of TF-eQTLs were observed in more than one tissue. (TIF) [file pgen.1009719.s007.tif]

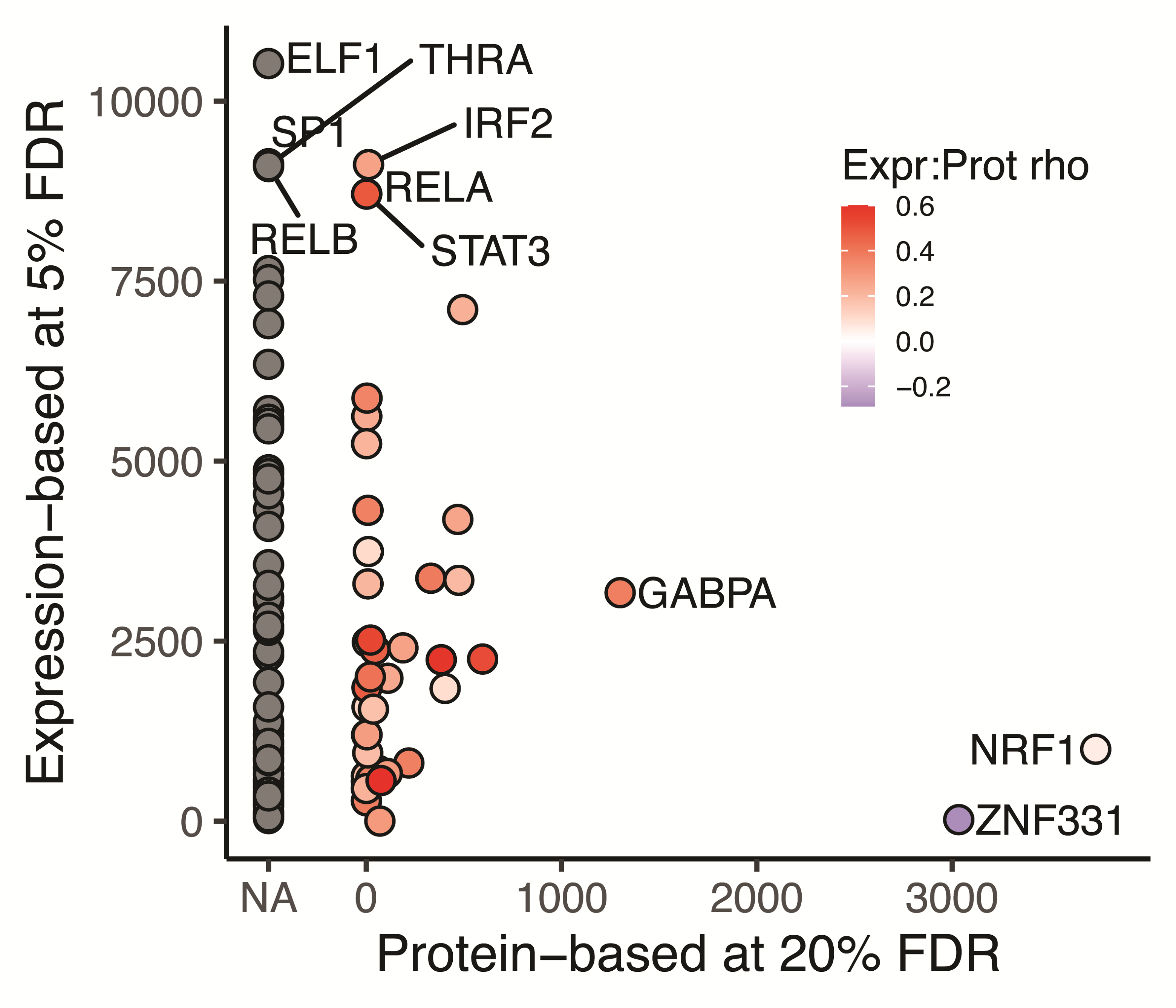

Supplement: S8 Fig — Number of cross-tissue expression-based TF-eQTLs are plotted versus number of cross-tissue protein-based TF-eQTLs. Dots are colored by Spearman correlation of cross-tissue median TF protein and expression levels. (TIF) [file pgen.1009719.s008.tif]

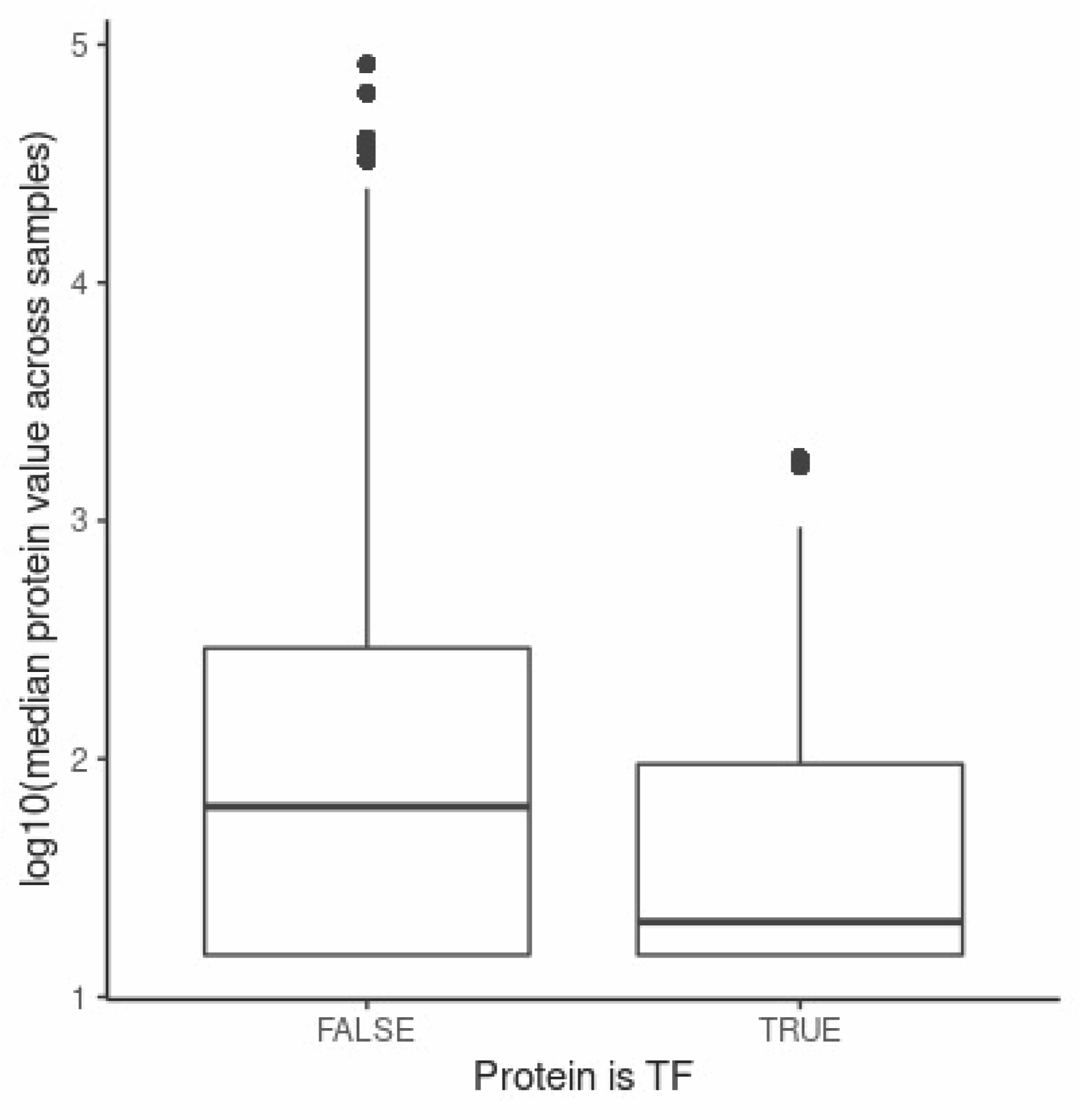

Supplement: S9 Fig — Log10 of median relative protein abundance for all measured proteins, subset by whether the protein is a tested TF or not. (TIF) [file pgen.1009719.s009.tif]

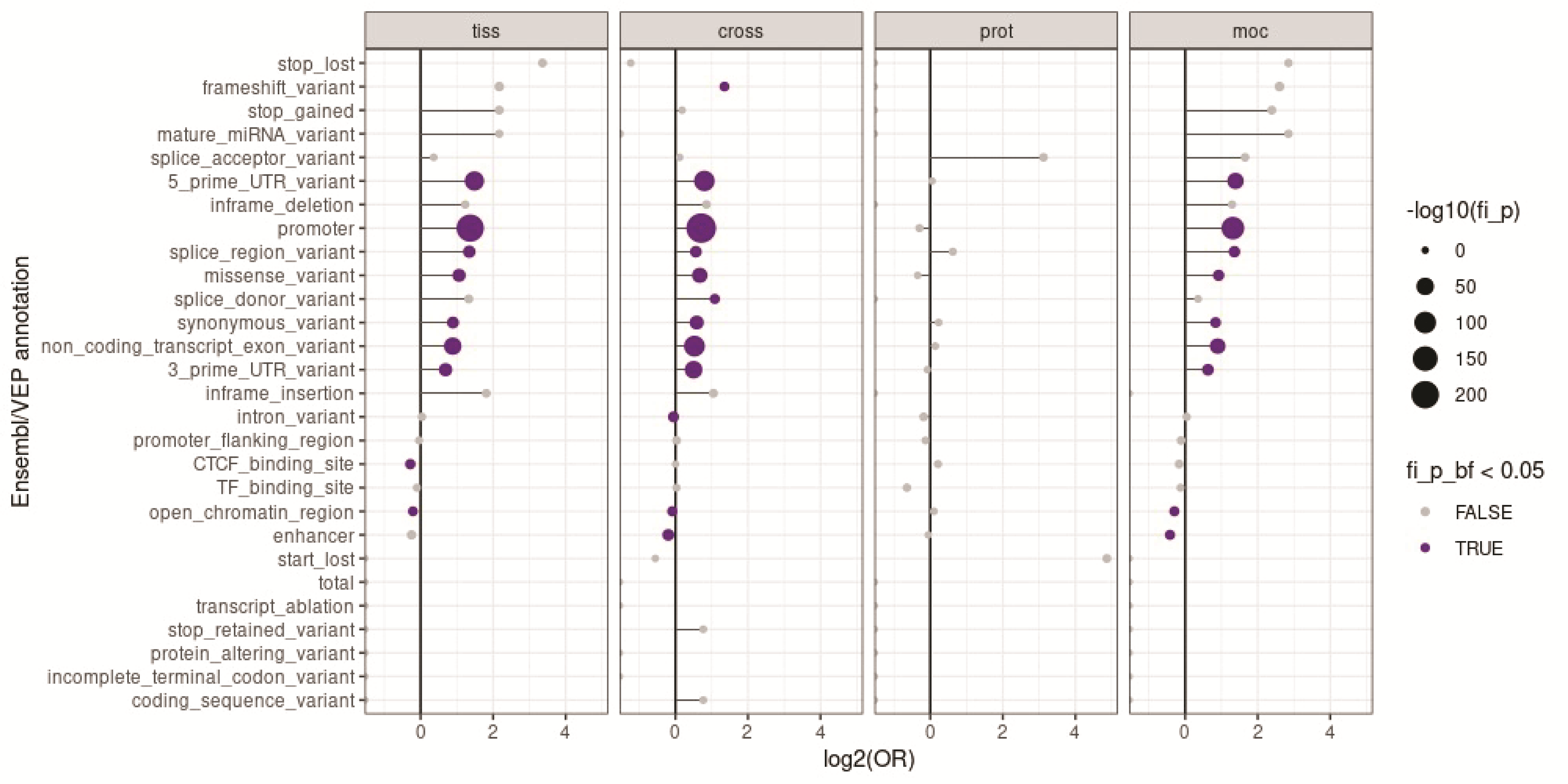

Supplement: S10 Fig — Enrichment of top TF-eQTL variant in each dataset for various genomic annotations from Ensembl Variant Effect Predictor. (TIF) [file pgen.1009719.s010.tif]

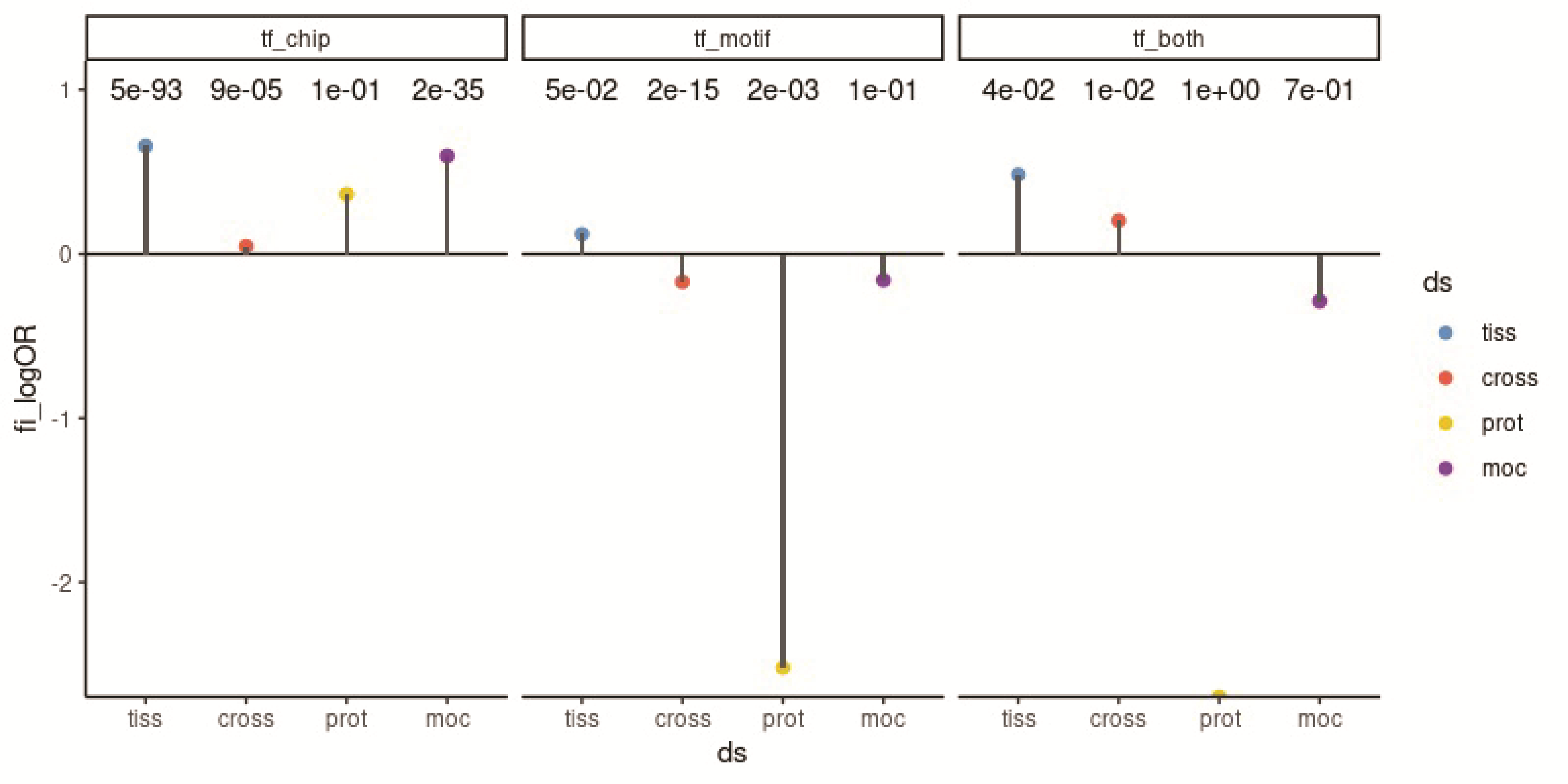

Supplement: S11 Fig — Enrichment of top TF-eQTL variant in each dataset for TF overlap, as defined by ENCODE TF ChIPseq peaks, HOCOMOCO predicted motifs, or both annotations together. Fisher’s exact test p-values are plotted. (TIF) [file pgen.1009719.s011.tif]

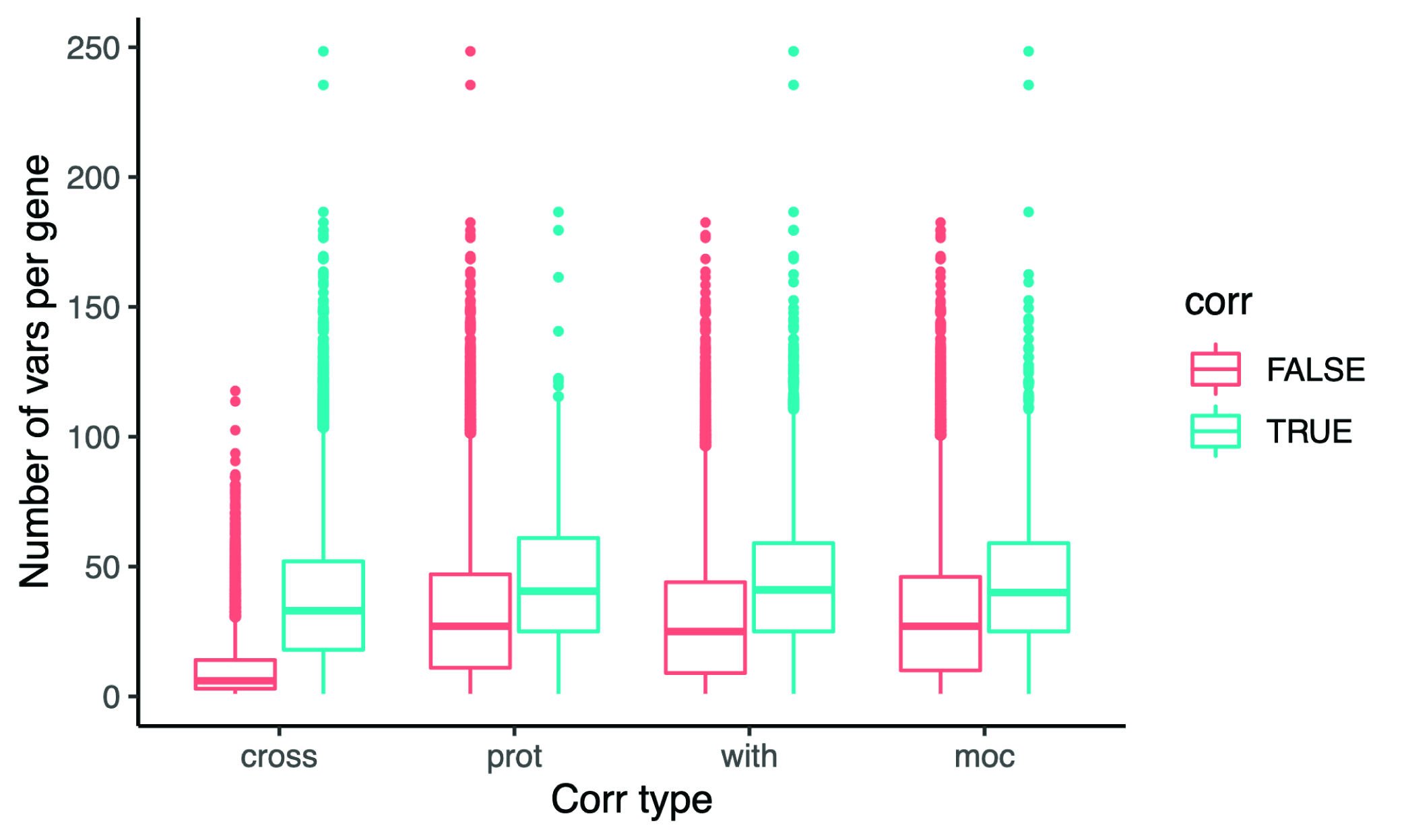

Supplement: S12 Fig — Number of tested variants per gene, separated by whether the gene had a TF-eQTL (color) in the given dataset (x-axis). Significant TF-eQTL genes tended to have more tested variants per gene than unsignificant genes. (TIF) [file pgen.1009719.s012.tif]

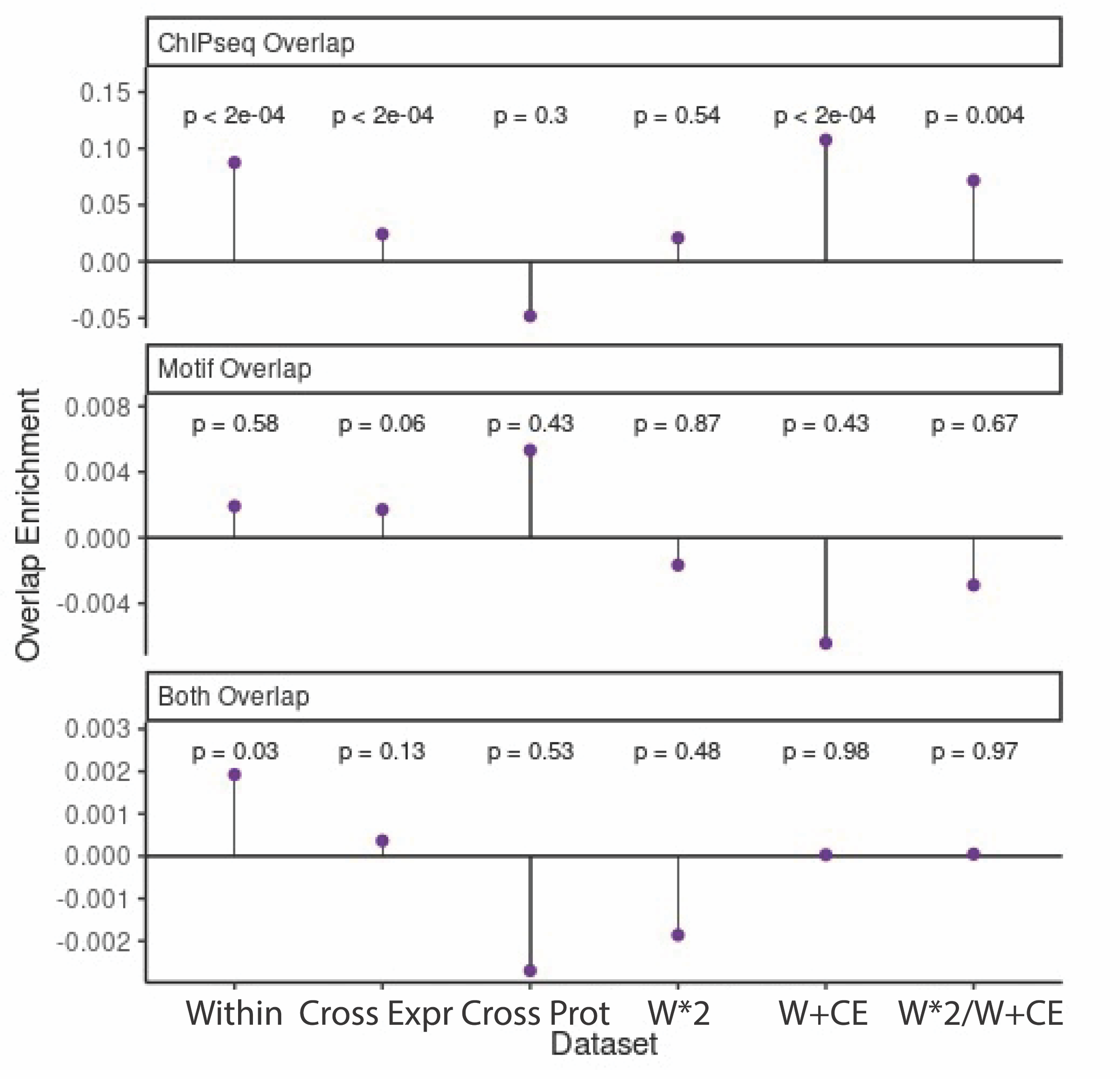

Supplement: S13 Fig — Overlap enrichment for each TF-eQTL dataset for ChIPseq overlap, motif overlap, and both overlap. (TIF) [file pgen.1009719.s013.tif]

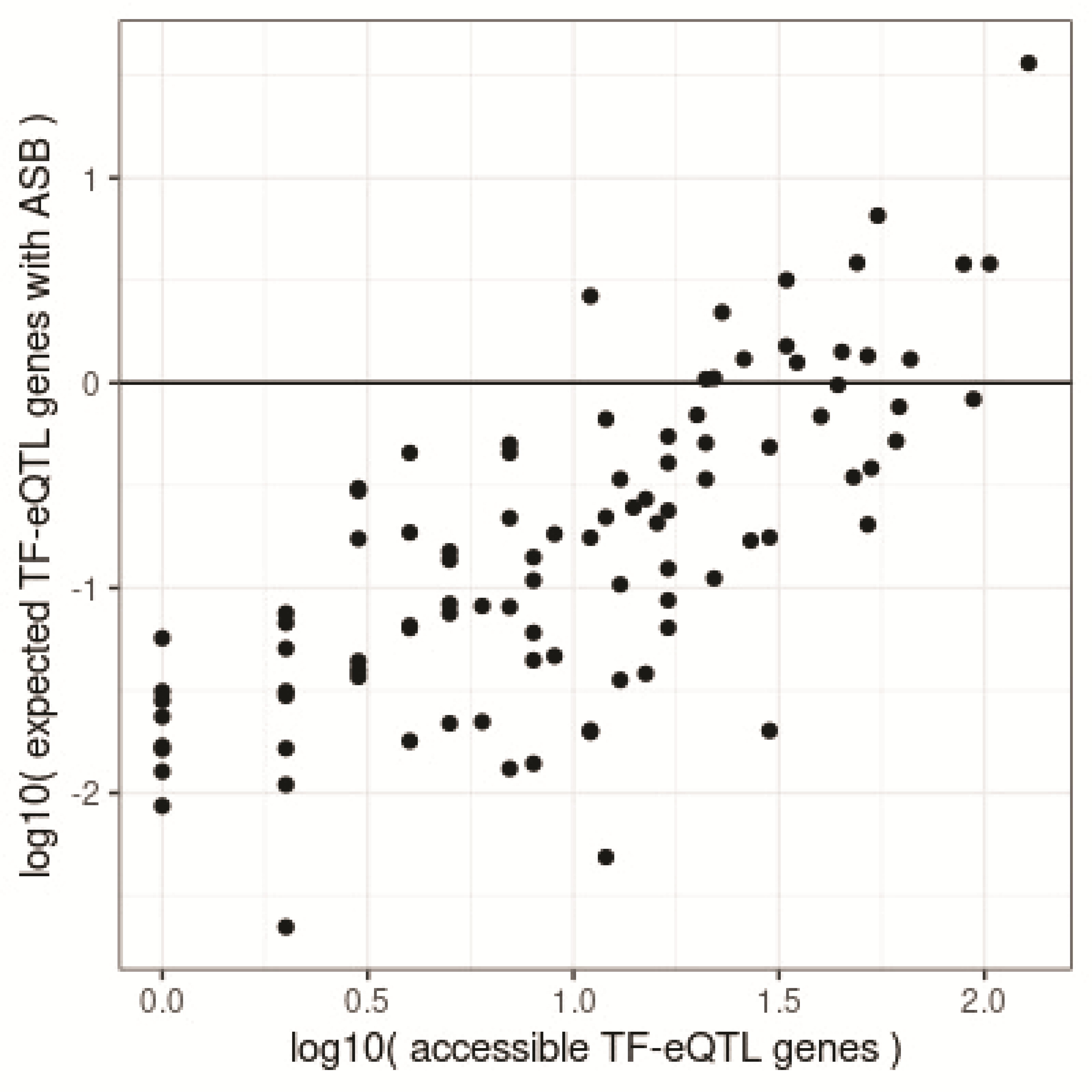

Supplement: S14 Fig — Number of expected TF-eQTL genes with ASB calculated by number of TF-eQTL gene variants accessible for a TF times percent of all accessible variants with ASB for that TF. (TIF) [file pgen.1009719.s014.tif]

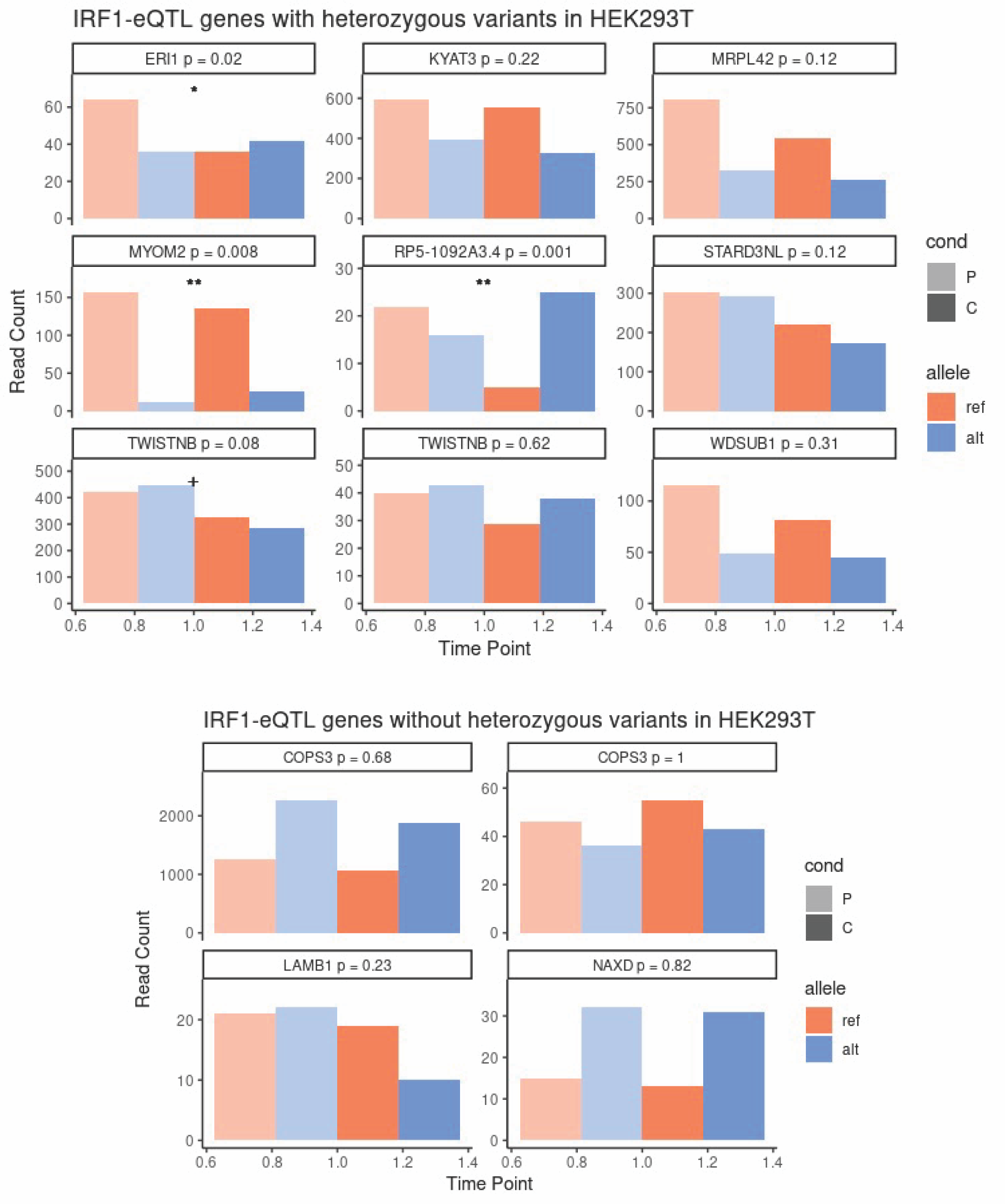

Supplement: S15 Fig — Read counts are plotted for reference (orange) and alternative (blue) coding variants in IRF1-eQTL genes. Promoter-knockdown samples are in light shade and control samples are in dark shade. IRF1-eQTL genes may be heterozygous for a top IRF1-eQTL variant (top) or not (bottom). Fisher’s exact test p value for data combined across timepoints is displayed at the top of each plot, while significance for individual timepoints is denoted by symbols: + p<0.10, * p<0.05, ** p<0.01. We see that three genes with heterozygous IRF1-eQTL variants show imbalanced ASE across conditions (ERI1, MYOM2, RP5-1092A3.4), while no genes without a heterozygous IRF1-eQTL variant. (TIF) [file pgen.1009719.s015.tif]

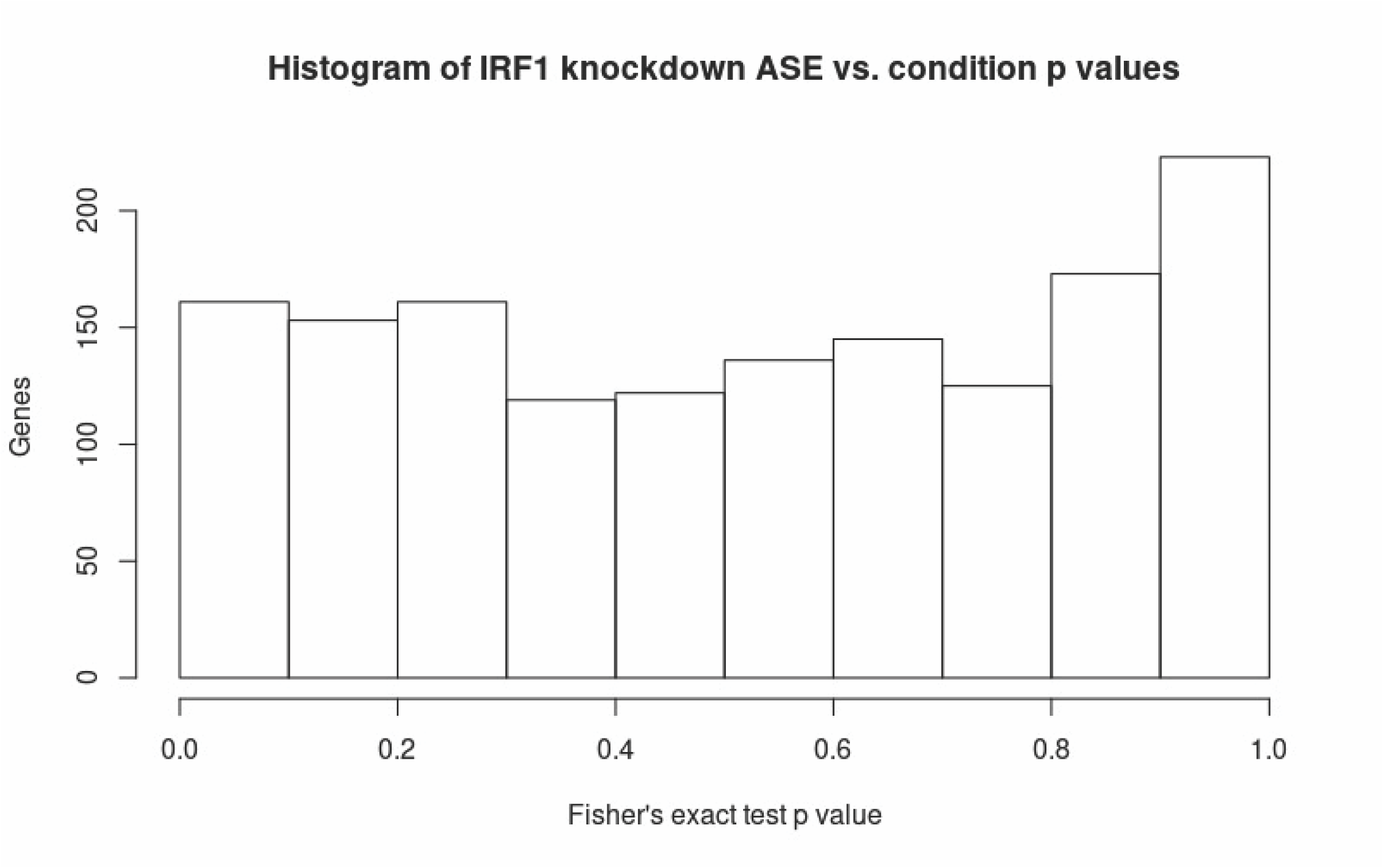

Supplement: S16 Fig — Fisher’s exact tests were run on 2x2 tables of allelic read counts in IRF1 knockdown and control experiments for all adequately covered genes. (TIF) [file pgen.1009719.s016.tif]

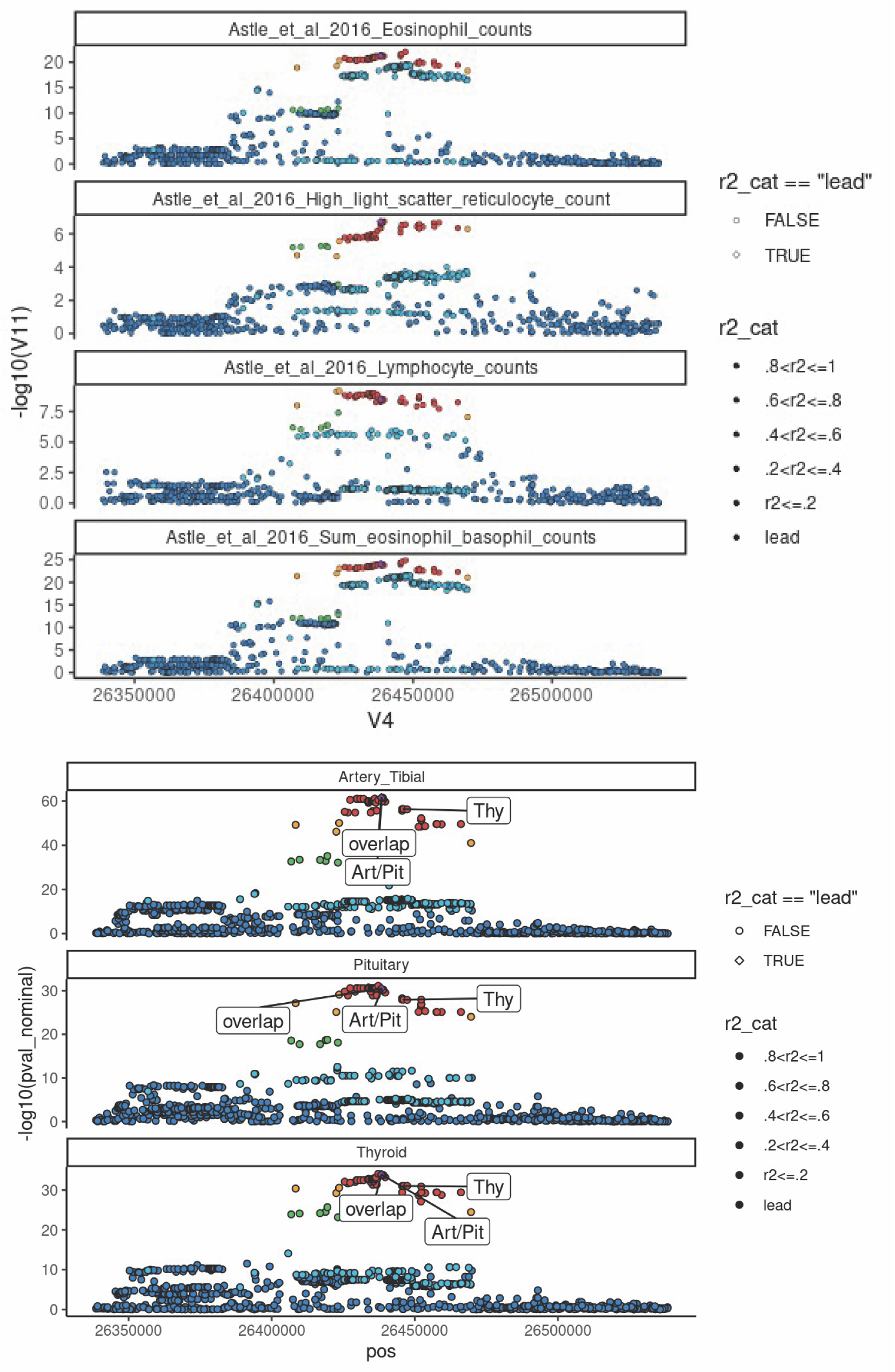

Supplement: S17 Fig — A) P values are plotted for four blood cell GWAS traits that colocalize with an APBB1IP eQTL in any tissue. B) P values are plotted for APBB1IP eQTL in three tissues with an IKZF1-eQTL signal. Top IKZF1-eQTL variants are labeled, as well as a variant that overlaps an IKZF1 motif and ChIP-seq peak (overlap). (TIF) [file pgen.1009719.s017.tif]

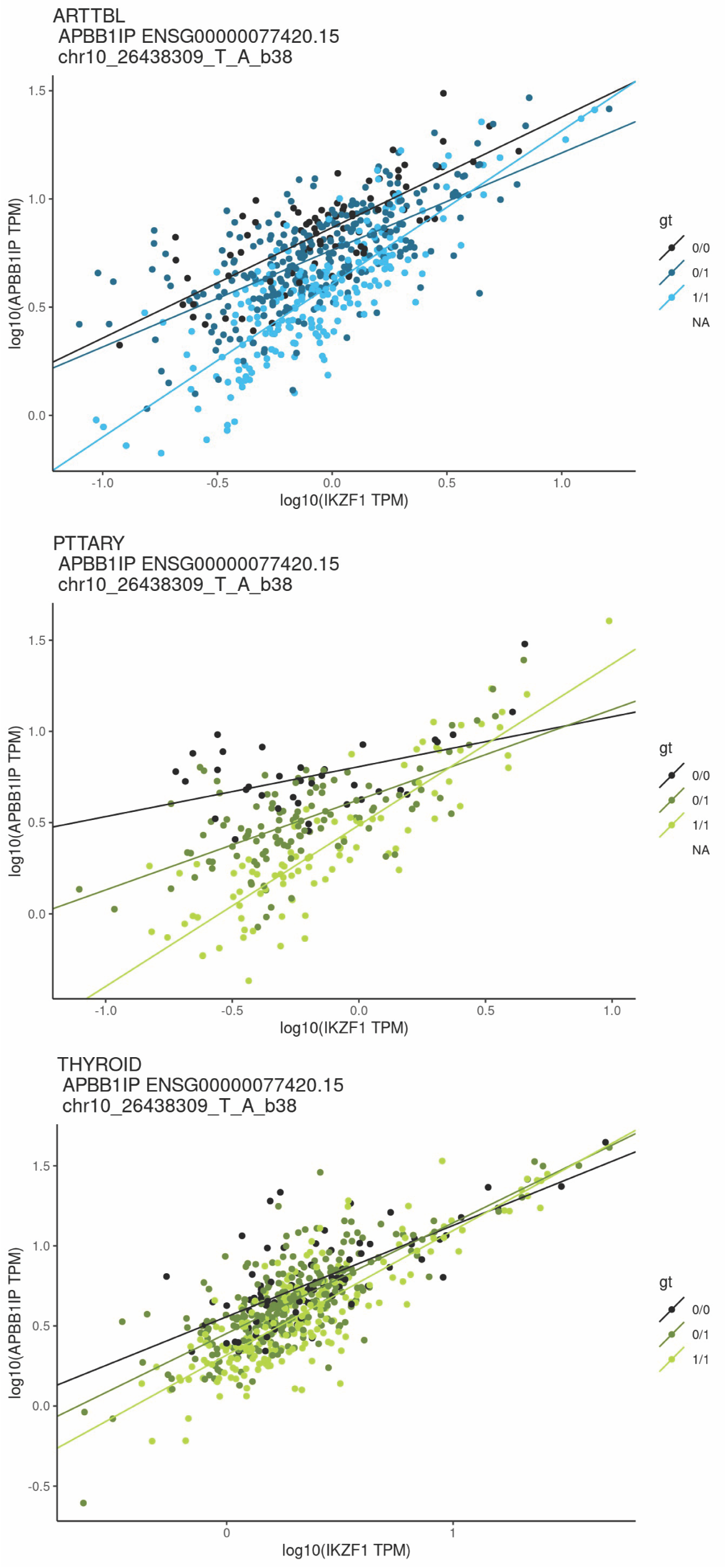

Supplement: S18 Fig — Individuals are plotted by APBB1IP expression vs. IKZF1 TF expression in three tissues. Trend lines show linear regression lines per genotype. All three tissues had a significant IKZF1-eQTL. (TIF) [file pgen.1009719.s018.tif]

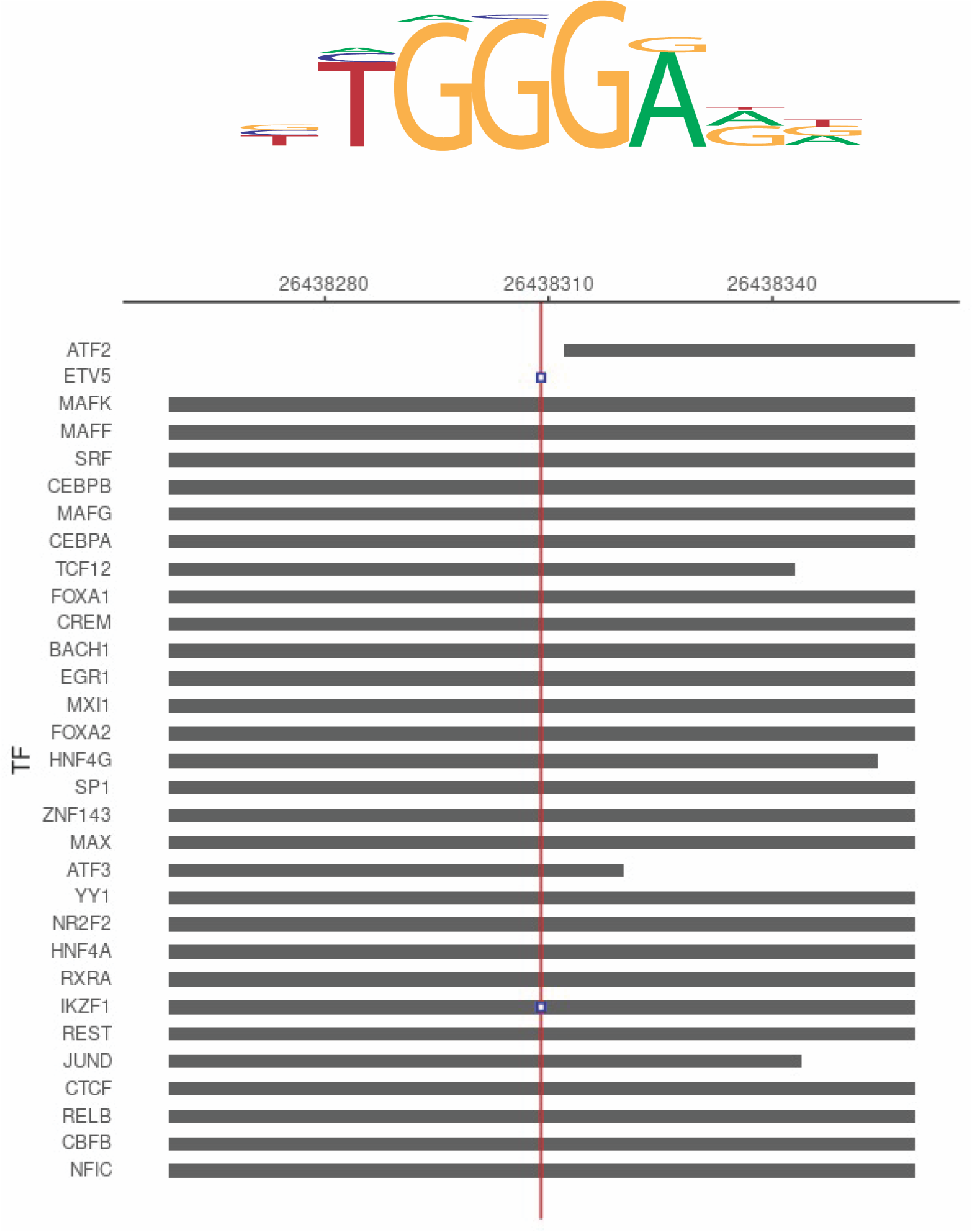

Supplement: S19 Fig — rs1335540 (red line) overlaps multiple TF ChIPseq peaks and two TF motifs. IKZF1 is the only TF which the SNP overlaps both of. The IKZF1 motif logo is displayed at the top of the figure, as calculated in HOCOMOCO v11 [50]. (TIF) [file pgen.1009719.s019.tif]
