# Supplementary material for: Transcription factor regulation of eQTL activity across individuals and tissues
Source: PLoS Genet. 2022 Jan 31;18(1):e1009719. doi: 10.1371/journal.pgen.1009719 (PMC8830792; doi:10.1371/journal.pgen.1009719)
Supplement: Attachment — Submitted filename: Review.pdf [file pgen.1009719.s025.pdf]

The manuscript from Flynn et al. utilizes publicly available data to identify interactions between transcription factor (TF) expression/binding and eQTLs. The authors primarily leverage the GTEx dataset - an initiative led in part by the lead author Dr. Lappalainen. The authors identify a number of eQTLs with effect sizes correlated with TF expression either across individuals/within a tissue or across tissues. They also perform similar analyses with protein levels of TFs - although these were less fruitful. The authors characterize the degree of sharing and genomic features of TF-eQTLs. The authors use a previously published CRISPRi experiment to validate the findings of eQTL interactions with IRF1, and compare use a published dataset of GxE eQTLs to demonstrate how TF-eQTLs may be driving contex-specific QTLs. Finally, the authors overlap TF-eQTLs with GWAS variants to demonstrate how TF-eQTLs may contribute to complex traits. Overall the findings of this study are of broad interest to the genomics community - particularly the functional genomics community. The authors do a reasonable job of contextualizing these results and the methods are well described - along with the github code being publicly available. I have no major concerns with this manuscript in the current form, but have a small number of questions/comments that I suggest the authors address.

1) FDR. The “most” major of my comments is around FDR. The authors vary FDR cut offs for significance depending on the analysis - e.g. using 20% for the first analysis of TF-eQTLs within tissues (line 143) and then 5% across tissues (line 174). The authors should either select a single cut off OR justify why it is appropriate to vary these cutoffs.
2) I’m a bit confused by the X cell results. It seems strange that one of the tissues with the highest variance in cell-type composition between individuals is cultured fibroblasts. These cultures should be relatively pure as fibroblasts grow very rapidly and robustly. Additionally, the variable cell-type Th2 doesn’t make a lot of sense to me - particularly not at a mean frequency of 25% (if I’m reading the X axis of Fig. S5 correctly).
3) On lines 162 and in Fig. 2C I’m not sure what negative enrichment means. Is this a depletion? I also found that Fig. 2C was a bit hard to interpret in the context of the text. The two rows at the top of the heatmap are not explained - these are also the only places where a blue (negative enrichment) square was found. I’m not sure how to link what is said around line 162 to what I am seeing in Fig. 2C.
4) Somewhat pedantic, but in the manuscript you move from Fig. 2A to Fig. 2C and do not include Fig. 2B until later in the paper.
5) On line 164 the authors reference the clustering of within tissue TF-eQTL relations looks similar to tissue gene expression, but they do not show clustering data for TF-eQTLs - Sfig. 3 only shows expression data.
6) The only place the methods and text where a bit unclear was when the authors describe the SNP set for the aFC analysis. The authors state in the main text they use the “filtered variant-gene pair” and in the methods they state they use “all eVariant-eGene pairs”. In the main text the authors also state “by ignoring eQTL significance cutoffs”. It was not clear what significance cutoff was ignored - the initial eQTL significe? Some clarity here would be helpful!
7) In the annotation and TF-binding of TF-eQTL interactions section the authors describe how TF-eQTLs are more likely to overlap a TFBS of their interacting TF, but also state they fall into the regulon of that TF. It wasn’t clear how regulons were defined.
8) Lastly the authors use TF-eQTL throughout the text and figures, but the meaning of this changes somewhat depending on the section - e.g. in Fig. 5 TF-eQTL actually means dual evidence TF-eQTL while in other sections it means only cross tissue or only within tissue etc.
