# Supplementary material for: Transcription factor regulation of eQTL activity across individuals and tissues
Source: PLoS Genet. 2022 Jan 31;18(1):e1009719. doi: 10.1371/journal.pgen.1009719 (PMC8830792; doi:10.1371/journal.pgen.1009719)
Supplement: Attachment — Submitted filename: Reviewer responses.pdf [file pgen.1009719.s026.pdf]

Reviewer #1:

The manuscript from Flynn et al. utilizes publicly available data to identify interactions between transcription factor (TF) expression/binding and eQTLs. The authors primarily leverage the GTEx dataset - an initiative led in part by the lead author Dr. Lappalainen. The authors identify a number of eQTLs with effect sizes correlated with TF expression either across individuals/within a tissue or across tissues. They also perform similar analyses with protein levels of TFs - although these were less fruitful. The authors characterize the degree of sharing and genomic features of TF-eQTLs. The authors use a previously published CRISPRi experiment to validate the findings of eQTL interactions with IRF1, and compare use a published dataset of GxE eQTLs to demonstrate how TF-eQTLs may be driving context-specific QTLs. Finally, the authors overlap TF-eQTLs with GWAS variants to demonstrate how TF-eQTLs may contribute to complex traits. Overall the findings of this study are of broad interest to the genomics community - particularly the functional genomics community. The authors do a reasonable job of contextualizing these results and the methods are well described - along with the github code being publicly available. I have no major concerns with this manuscript in the current form, but have a small number of questions/comments that I suggest the authors address.

RE: We appreciate the reviewer's helpful comments and concerns. As detailed below, we have re-done analyses and added the requested analyses as needed.

1) FDR. The "most" major of my comments is around FDR. The authors vary FDR cut offs for significance depending on the analysis - e.g. using 20% for the first analysis of TF-eQTLs within tissues (line 143) and then 5% across tissues (line 174). The authors should either select a single cut off OR justify why it is appropriate to vary these cutoffs.

We have updated the analysis to use a 5% FDR threshold for the within-tissue correlations and cross-tissue protein-based correlations. Additionally, we updated our within-tissue multiple testing correction to be more consistent with the cross-tissue correlation corrections, which explains why we were now able to find more within-tissue TF-eQTLs at the 5% FDR threshold. The overall results and conclusions stay the same.

2) I'm a bit confused by the X cell results. It seems strange that one of the tissues with the highest variance in cell-type composition between individuals is cultured fibroblasts. These cultures should be relatively pure as fibroblasts grow very rapidly and robustly. Additionally, the variable cell-type Th2 doesn't make a lot of sense to me - particularly not at a mean frequency of 25% (if I'm reading the X axis of Fig. S5 correctly).

In fact, GTEx fibroblast cell lines are quite variable between individuals, as we showed in our previous analysis of cell type composition of GTEx samples (Kim-Hellmuth et al. 2020 Science). This may be partially due to the fact that the samples are from post-mortem donors.

XCell enrichment cannot be interpreted as a proportion of a given cell type. It is based on enrichment of the target gene set expression in the samples, and it does not always capture cell

types correctly since the reference sets do not include all cell types present in GTEx tissue samples. We agree that the Th2 cells are probably not present in the fibroblast samples, but the fact that enrichment is so variable across samples does suggest that the cell type composition varies.

3) On lines 162 and in Fig. 2C I'm not sure what negative enrichment means. Is this a depletion? I also found that Fig. 2C was a bit hard to interpret in the context of the text. The two rows at the top of the heatmap are not explained - these are also the only places where a blue (negative enrichment) square was found. I'm not sure how to link what is said around line 162 to what I am seeing in Fig. 2C.

By negative "negative enrichment" we indeed mean "depletion" -- this has been corrected. We have clarified the Fig 2C caption to make it easier to interpret. It is discussed in multiple places: first when discussing the within-tissue TF-eQTLs (lines 173-179), and then when discussing the cross-tissue TF-eQTLs and comparing them to within-tissue TF-eQTLs (lines 203-206, 211-213). We have updated the text at lines 173-179 (formerly line 162) to be more clear.

4) Somewhat pedantic, but in the manuscript you move from Fig. 2A to Fig. 2C and do not include Fig. 2B until later in the paper.

We agree that this is not ideal, but Figure 2C uses results from 2A and 2B so we would like to keep it in the current order.

5) On line 164 the authors reference the clustering of within tissue TF-eQTL relations looks similar to tissue gene expression, but they do not show clustering data for TF-eQTLs - Sfig. 3 only shows expression data.

The clustering of TF-eQTL relations across tissues is shown in Fig 2C (which we refer to in the text), and SFig. 3 is provided to show the gene expression clustering.

6) The only place the methods and text where a bit unclear was when the authors describe the SNP set for the aFC analysis. The authors state in the main text they use the "filtered variant-gene pair" and in the methods they state they use "all eVariant-eGene pairs". In the main text the authors also state "by ignoring eQTL significance cutoffs". It was not clear what significance cutoff was ignored - the initial eQTL significe? Some clarity here would be helpful!

This was indeed slightly unclear, and we have revised the corresponding text accordingly. All analyses were done only with the 1,032,124 filtered eVariant-eGene pairs. As for the eQTL significance cutoffs, you are correct that we are referring to the initial eQTL significance. We included the calculated eQTL effect size in our analysis even if the initial eQTL in that tissue was not significant.

7) In the annotation and TF-binding of TF-eQTL interactions section the authors describe how TF-eQTLs are more likely to overlap a TFBS of their interacting TF, but also state they fall into the regulon of that TF. It wasn't clear how regulons were defined.
The TFBS overlap enrichment was calculated on the specific variants that were tested for the TF-eQTL, while the regulon enrichment was calculated for the gene of the TF-eQTL, using regulon sets defined in (Garcia-Alonso et al. 2019). We have added more detail on regulon sets to the Methods section to make this more clear.

8) Lastly the authors use TF-eQTL throughout the text and figures, but the meaning of this changes somewhat depending on the section - e.g. in Fig. 5 TF-eQTL actually means dual evidence TF-eQTL while in other sections it means only cross tissue or only within tissue etc.

Thank you for this comment -- we have updated the text to ensure that we refer to dual-evidence (DE) TF-eQTLs when necessary.

Reviewer #2:

Extensive efforts have catalogued the regulatory architecture of gene expression variation across the human body, but less is known about the mechanisms associated with variation across other contexts. In the present study, Flynn, et al. propose the use of TF expression and eQTL effect size to identify putative upstream TF regulators of eQTL effects. This work represents a creative analysis approach by using the p-value from a [TF]*genotype interaction term in a regression framework and re-use of several publicly available data sets including GTEx, ENCODE, and a CRISPRi perturbation study. While the proposed method and its overall goals are of great interest, there are concerns with its current implementation.

RE: We appreciate the reviewer's helpful comments and concerns. We have addressed their first major point by applying a single 5% FDR cutoff to each TF-eQTL dataset, and we have clarified the text and performed additional analyses to address their remaining points.

Major points:

1. It is stated in the methods that a 20% FDR is used on the within-tissue TF-eQTL analysis because additional filtering was performed. Other than removing tissues with high cell type composition variability and the annotation overlap filtering, which left only 7% of 5% MAF SNPs in the list, what other filtering was performed? How many results survive a more conservative FDR threshold? Presenting and focusing on only the lenient 20% FDR results leaves one a bit skeptical.

We have updated the analysis to use a 5% FDR threshold for the within-tissue correlations and cross-tissue protein-based correlations. We updated our within-tissue multiple testing correction to

be more consistent with the cross-tissue correlation corrections, which explains why we were now able to find more within-tissue TF-eQTLs at the 5% FDR threshold. The overall results and conclusions remain unchanged.

2. The authors select eQTL signal variants using an intersection between genetic fine-mapped, ChIP-seq peak overlapping, and TF motif overlapping variants. How does this set of SNPs compare the lead SNP (or highest posterior probability fine-mapped SNP) at the signal? Is the lead or a very high LD SNP always selected? Also, why was a functional fine-mapping approach not used, as this seems like a natural implementation of the set operations that were performed?

Thank you for this thoughtful question. We found that the lead tissue eQTL variant was included in our filtered variant set approximately 20% of the time, while 93% of all eQTL genes were included in our filtered set. We have added this information to the text and supplementary figures.

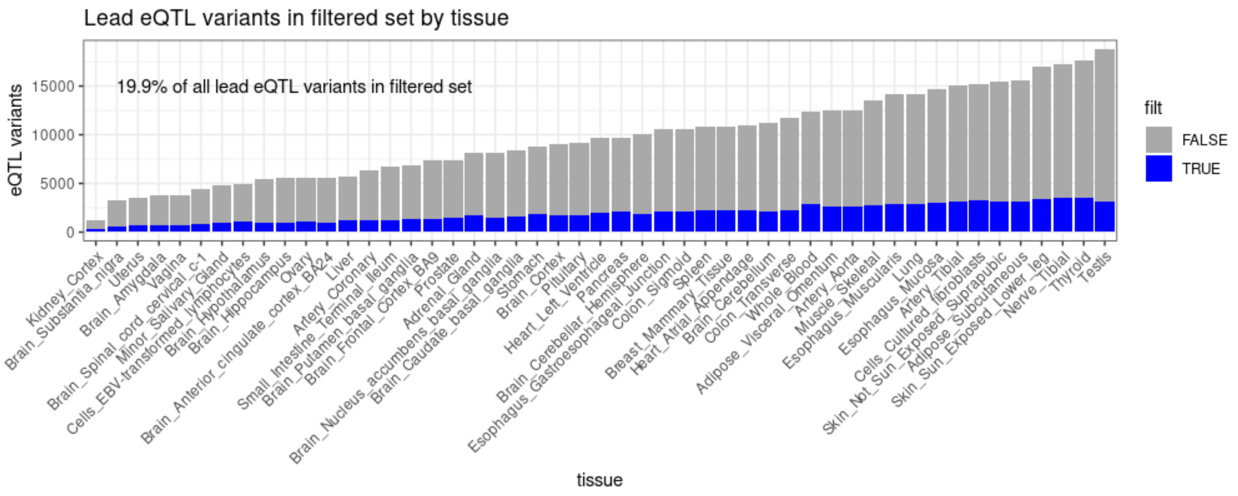

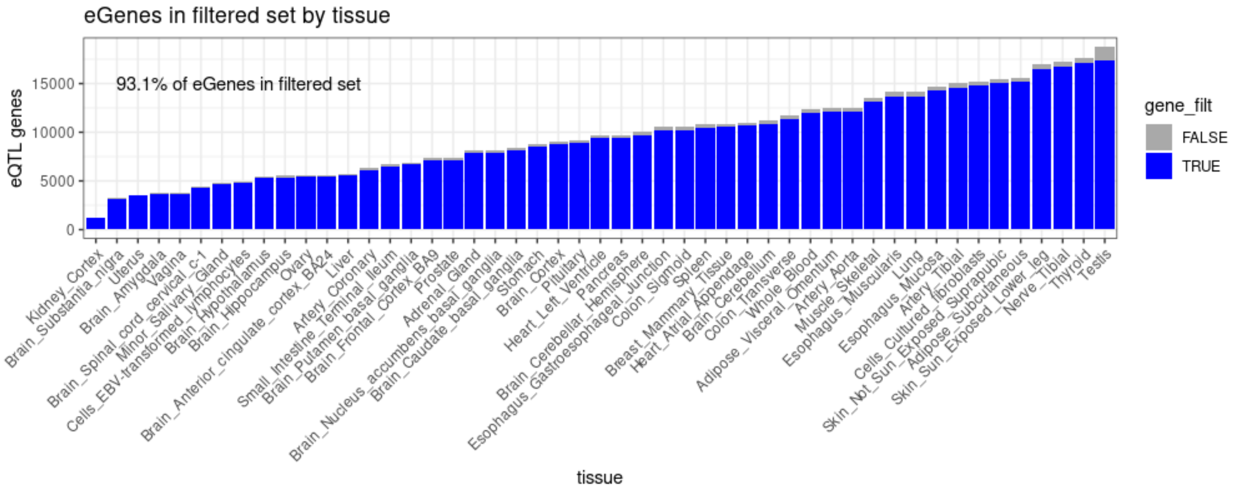

Early in the project, we tested DAP-G, Caviar, and CaVEMaN fine-mapping tools, and selected Caviar for the final analysis. While functional fine-mapping would have had benefits, we were concerned that it could introduce subtle biases in how variants are selected with respect to

functional elements and greatly complicate some of the downstream enrichment analyses. With a fine-mapping approach that uses association signal and LD only, we may lose some power but are unbiased with respect to annotations.

3. There appear to be several circular analyses performed for enrichment testing of the discovered signals.

We appreciate the reviewer's concern, and we have updated the text to be clear that these analyses were all performed with the correct background sets, as detailed below.

a. First, the approach used to test whether TF-eQTL interactions overlapped with TFBSs: given that the initial set of variants was filtered to only those that overlapped with one or more TF ChIP-seq peak and were present in a HOCOMOCO motif, one expects these variants to be enriched for those annotations. The authors should take this initial filtering approach into account when performing enrichment analyses.

Our analysis did take this into account. The background set of variants used for ChIPseq and motif enrichments was only the "filtered variants,", i.e., all variants that were fine-mapped and overlapped a ChIPseq peak and TFBS motifs. We have updated the methods to make sure this detail is clear.

b. Second, a similar circularity appears to happen for the GWAS colocalization enrichment analysis of the TF-eQTL signals. Again, initial filtering was performed for eQTL that overlap one or more TF ChIP-seq peak and were present in a HOCOMOCO motif. Thus, the entire eQTL list is not the appropriate null to compare for GWAS colocalization. Instead, the authors should look at the eQTL signals they tested after filtering and perhaps compare to see if there is a significant enrichment difference between the signals they report as significant versus the signals they tested but did not report as significant.

This was taken into account. Only eQTLs that were tested in our analysis (i.e. had at least one filtered eQTL variant) were used for the background set in enrichment testing. We have updated the methods to make sure this detail is clear.

Minor points:

1. What is the overall similarity between the motifs of TFs tested, and what was the reasoning for using collapsing PWMs and using regex matching for motif analysis versus a more traditional PWM scanning approach?

Based on motif clustering results from other sources, we see that our tested TF motifs cover a range of sequence space. We examined 145 of our tested TF HOCOMOCO v11 motifs in a clustering analysis performed by Dr. Jeff Viestra (https://www.vierstra.org/resources/motif_clustering), and we found that these TF motifs clustered into 80 groups, with 1 to 11 TFs per cluster.
We agree that using a PWM scanning approach may have resulted in stronger motif overlap enrichments. However, using a PWM scanning approach would require us to set binding score thresholds for each transcription factor motif, each of which had different information content. To

avoid this difficult problem, we chose a binary match/no match option with the IUPAC degenerate motif. We are planning to revisit this question with a novel PWM scanning approach developed by our collaborators.

2. Also related to the PWMs, in the methods section, it's not clear what "trimming any less confident bases (lowercase letters) from the ends of the sequence" really means. What are the actual threshold used to determine less confident bases and ends of the sequence?

We have updated the text to clarify this. The lowercase letters in the HOCOMOCO motif refer to bases that are degenerate to three (bdhv) or four nucleotides (n), while the uppercase letters refer to bases that match one (ACGT) or two (RYSWKM) nucleotides. Because many of the motifs were rather long (up to 24 bases), we removed bases from the ends of the motifs until we reached a position with only one or two possible nucleotides.

3. There are two SFigure 10s referenced in the text and in the supplement.

Thank you for bringing this to our attention. We have corrected the SFigure labels.
